# In Situ Facile Synthesis of Low-Cost Biogenic Eggshell-Derived Nanohydroxyapatite/Chitosan Biocomposites for Orthopedic Implant Applications

**DOI:** 10.3390/nano12234302

**Published:** 2022-12-04

**Authors:** Sankar Sekar, Sejoon Lee

**Affiliations:** 1 Department of Semiconductor Science, Dongguk University-Seoul, Seoul 04620, Republic of Korea; 2 Quantum-Functional Semiconductor Research Center, Dongguk University-Seoul, Seoul 04620, Republic of Korea

**Keywords:** biocomposite, implant coating, indentation, biocompatibility

## Abstract

In situ facile synthesis and the characterization of nanohydroxyapatite/chitosan (nHAP/CS) biocomposites were investigated for examining their potential applications in orthopedic implant technology. Firstly, the bare nHAP, europium-doped hydroxyapatite (Eu-nHAP), yttrium-doped hydroxyapatite (Y-nHAP), and Eu- and Y-codoped hydroxyapatite (Eu,Y-nHAP) nanoparticles were synthesized by the wet precipitation technique using biowaste-eggshell-derived calcium oxide powders. Then, through ultrasonication using the nanohydroxyapatite/chitosan mixtures (molar ratio = 1:2), the nHAP/CS, Eu-nHAP/CS, Y-nHAP/CS, and Eu,Y-nHAP/CS biocomposites were fabricated. Among them, Eu,Y-nHAP/CS showed higher cell viability (94.9%), higher solubility (pH = 7.6 after 21 days), and greater antibacterial activity than those of the other composites. In addition, Eu,Y-nHAP/CS exhibited improved mechanical properties compared with the other composites. For example, the nanoindentation test displayed the Eu,Y-nHAP/CS-coated 316L stainless steel implant to possess a higher Young’s modulus value (9.24 GPa) and greater hardness value (300.71 MPa) than those of the others. The results indicate that the biomass-eggshell-derived Eu,Y-doped nHAP is of good use for orthopedic implant applications.

## 1. Introduction

The rise of scientific knowledge in nanofabrication has altered the materials in science and technology for materializing various emerging biomaterials, which can imitate the structure and the morphology of biotic tissues [1,2,3]. However, varieties of biomaterials that can repair and/or replace various diseased or damaged body parts are still lacking. In general, the interactions between the artificial implants and the surrounding bone tissues are determined by the surface properties of the implants [4]. The apatite of natural bone is nonstoichiometric hydroxyapatite because the bone contains various elements with different concentrations [5]. The long-term interaction of the stable synthetic implant in the surrounding tissues minimizes the ablation of post-operative implant failures, which may occur due to aseptic loosening and infections [6]. In addition, the osteogenic coatings on the implant surface facilitate protein attachments, which may stimulate osteoblast proliferation in an extracellular matrix [7]. Because of the inorganic phase similarity with natural bones, hydroxyapatite and its related composites have been extensively studied in bone tissue engineering for improving both the repair and regeneration of bones during implantation and regenerative medicine [8]. Owing to their excellent biocompatibility with enhanced physicochemical and biological properties, alternative biomaterials are widely used in orthopedic and dental implant applications [9]. Among the various alternatives, hydroxyapatite (HAP, Ca_10_(PO_4_)_6_(OH)_2_) is one of the major bioceramics present in the form of nanocrystallites in human bones and teeth [10]. Furthermore, HAP can lead to bone ingrowth and create osteoconductive bonds to bones because of its better osteoconductivity and enhanced biocompatibility [11]. Therefore, extensive studies on HAP have been conducted for developing orthopedic and dental prostheses [12,13]. During the last two decades, biowaste-derived HAP has been widely studied because of its cost-effectiveness and environmental friendliness compared with conventional synthetic procedures. For instance, eggshells [14,15], fish bone [16,17], bovine bone [18,19], and crab shells [12,20] are typical examples that can be utilized for the facile synthesis of biowaste-derived HAP. Unfortunately, however, the high brittleness and the low mechanical strength of bare HAP somewhat restrict its long-term clinical application. Thus, the “surface coating method” has been proposed and demonstrated for improving implant durability [21,22]. Additionally, the bioactivity of pure HAP is limited because of its low resorb ability. Namely, pure HAP somewhat restricts bone formation and resorption because of its unusually high affinity with bone tissue. To solve this issue, the ionic substitution of the HAP lattices was conceived and investigated [23]. Generally, human bone is nonstoichiometric because of various elemental constituents, such as Na, Mg, K, Sr, Nd, Eu, La, Ga, Zn, Ba, Cu, Al, Fe, F, Cl, and Si. Accordingly, many recent studies have shown that the doping of mineral ions into HAP could induce the deposition of calcium and phosphate ions in physiological solutions, and it eventually improved cell viability and proliferation [24,25]. Furthermore, incorporating anionic and cationic substituents into HAP has also been reported as an effective way to modify the biological properties of HAP [26,27,28]. According to the literature [29,30,31,32], Y acts as a good dopant material for orthopedic applications because of its potential to increase conductivity as well as hydrophilicity. Moreover, Y containing biomaterials has exhibited high stability in various environments, and it could help to augment osteoblast adhesion during implantation. Likewise, Eu^3+^ enhances biological activity because of its low toxicity as well as constant luminescence compared with other rare-earth elements. Furthermore, the incorporation of the optimal Eu^3+^ content in the hydroxyapatite structure could improve the biological properties with no toxic effects. Additionally, an Eu^3+^ substitution in HAP could improve the osteogenic behavior of the composites, and it would eventually help promote new bone regeneration.

Meanwhile, the insufficient fracture toughness of HAP still has restricted its clinical applications, even though the aforementioned doping techniques allow for the enhancement of HAP’s performance. To improve its mechanical strength with reduced brittleness and uniform surface coating, very recently, adding biologically active non-toxic polymers into HAP was suggested by Oner and coworkers [33]. Additionally, synthetic biopolymers have been considered as a promising biomaterial because of their enhanced biocompatibility in biological environments [34,35,36]. Along with its enhanced biocompatibility, the low production cost, high corrosion resistance, and high chemical stability of HAP could also extend its applications toward various biomedical applications. Recently, it has been reported that various HAP/polymer composites could enhance biological behaviors [37,38]. For example, surface coating a mineral-substituted HAP onto a polymer-coated 316L stainless steel (SS) implant is one of the promising techniques that can prevent metal ion release during implantation in biological environments [39]. In addition, it was reported that a porous mineral-substituted HAP coating of the biopolymer-coated 316L SS implant surface could not only improve implant fixation but also reduce implant corrosion in physiological environments [40]. Another key characteristic of HAP is its mechanical stability, which can be examined by nanoindentation. Several studies have shown that the HAP-composite-coated implant would lose its mechanical integrity [41,42], and such a behavior relies on the ion type, particle size, and composition of the nanocomposites [43]. Additionally, the textural porosity and the structural regularity of the HAP nanocomposites also affect the mechanical strength [44].

All of the above background knowledge prompted us to investigate the effects of a mineral-substituted nano-hydroxyapatite (nHAP) coating on the mechanical strength, bioactivity, antibacterial activity, and cell culture properties of the 316L SS implant. In this study, we chose Eu and Y as the mineral substituents to modify HAP’s properties because of their improved bioactivity (e.g., cell proliferation and growth) and high biodegradability for potential biomedical applications [45]. The nHAP products were prepared by the facile wet precipitation method using biogenic eggshell-derived calcium oxide. During the wet precipitation process, Eu- and Y-doping were also performed using yttrium nitrate hexahydrate and europium nitrate pentahydrate precursors, respectively. Here, we note that, to verify the Eu- and Y-codoping effects, four different types of samples were prepared and assessed, i.e., (1) bare nHAP, (2) Eu-doped nHAP (Eu-nHAP), (3) Y-doped nHAP (Y-nHAP), and (4) Eu- and Y-codoped nHAP (Eu, Y-nHAP). In addition, it should be noted that chitosan (CS) was introduced into the resultant products to employ the advantage of the HAP/biopolymer composite system. Herein, the materials synthesis, materials characteristics, biological performances, and mineral doping effects are thoroughly examined and discussed in detail.

## 2. Materials and Methods

### 2.1. Materials

The biogenic eggshell wastes were collected from Namakkal, Tamil Nadu, India, and all the chemicals, yttrium nitrate hexahydrate (Y(NO_3_)_2_∙6H_2_O), europium nitrate pentahydrate (Eu(NO_3_)_3_∙5H_2_O), hydrochloric acid (HCl), sodium hydroxide (NaOH), cetyl trimethyl ammonium bromide (CTAB), chitosan (C_56_H_103_N_9_O_39_) and phosphoric acid (H_3_PO_4_), were purchased from Sigma Aldrich (St. Louis, MO, USA). The as-purchased high-purity analytical grade chemicals were used with no additional purification, and deionized (DI) water was used throughout all the necessary steps to avoid inorganic and organic contamination.

### 2.2. Synthesis of nHAP Samples

The collected eggshells were washed several times with DI water to remove the unwanted pretentious parts and dried at 80 °C for 4 h in a conventional air oven. Next, the dried eggshells were milled using a mortar to obtain a powdered type of eggshell. For the reduction of the eggshells’ calcium carbonate into calcium oxide (CaO), the obtained eggshell powder was heated at 800 °C for 2 h in a muffle furnace. Next, to obtain the aqueous solution, the CaO powder (0.4 M) was mixed into HCl (3 M) under vigorous stirring at 700–800 rpm for 4 h at room temperature. During this step, the aqueous CaCl_2_ solution could be obtained via the following chemical reaction:

(1) $$\mathrm{CaO}+2\mathrm{HCl}\to {\mathrm{CaCl}}_{2}\left(\mathrm{aq}.\right)+{\mathrm{CO}}_{2}+H_{2}O$$

After obtaining aqueous CaCl_2_, 0.2% CTAB was added into the CaCl_2_ solution and stirred for 1 h. Then, 0.3 M of H_3_PO_4_ was also added into the above CTAB-mixed CaCl_2_ solution under constant magnetic stirring. Thereafter, the pH level of the white mixture suspension was adjusted to 10 using 0.1 M of NaOH. Finally, the suspension was continuously stirred for 24 h to allow the chemical reaction for the synthesis of the nHAP precipitates. During this step, the precipitation of nHAP can be accomplished via the following chemical reaction:

(2) $$10{\mathrm{CaCl}}_{2}+6H_{3}{\mathrm{PO}}_{4}+20{\mathrm{NH}}_{4}\mathrm{OH}\to {\mathrm{Ca}}_{10}{\left({\mathrm{PO}}_{4}\right)}_{6}{\left(\mathrm{OH}\right)}_{2}↓+20{\mathrm{NH}}_{4}\mathrm{Cl}+18H$$

Similarly, the Eu-nHAP, Y-nHAP, and Eu,Y-nHAP products were obtained through the above sequences by only adding Y(NO_3_)_2_∙6H_2_O (0.05 M) and/or Eu(NO_3_)_3_∙5H_2_O (0.05 M) into the CaCl_2_ solution. After all the precipitation steps, the obtained precipitates were centrifuged and washed with DI water 5–7 times to remove the unreacted chemicals. Finally, the purified white precipitates were dried at 80 °C for 24 h and calcined at 800 °C for 4 h in a muffle furnace.

### 2.3. Fabrication of nHAP/CS Composite and nHAP/CS-Coated 316L SS Implant

The prepared nHAP nanoparticles were used for the nHAP/CS composite preparation. Firstly, the nHAP, Eu-nHAP, Y-nHAP, and Eu,Y-nHAP precipitates (1 g for each) were ultrasonically blended with the deacetylated CS (80%, 20 mg/mL) for 15 min with a pulse rate of 2 s. Here, we noted that the deacetylated CS was prepared by dissolving the CS polymers in acetic acid with a concentration of 5%. Then, the as-prepared nHAP/CS, Eu-nHAP/CS, Y-nHAP/CS, and Eu,Y-nHAP/CS composites were spin-coated at 2500 rpm for 3 min onto the surfaces of the 316L SS implant samples. We here note that, prior to the spin-coating process, the surfaces of the 316L SS implant samples were polished using silicon carbide grits (400–1200) to obtain their mirror surfaces. Finally, the spin-coated specimens were dried at room temperature and used for further characterization.

### 2.4. Materials Characterization

The functional groups of the nHAP, Eu-nHAP/CS, Y-nHAP/CS, and Eu,Y-nHAP/CS composites were analyzed through the KBr pellet technique with Fourier transform infrared spectroscopy (FTIR) using a Spectrum 100 system (PerkinElmer Inc., Waltham, MA, USA). To analyze the crystallographic properties of the samples, X-ray diffraction (XRD) measurements were conducted using a X’Pert PRO system (PANalytical, Almelo, the Netherlands) equipped with a CuKα radiation source (operating voltage: 40 kV and current: 30 mA). The surface topographies of the samples were analyzed with field emission scanning electron microscopy (FE-SEM) using a SIGMA VP system (ZEISS, Oberkochen, Germany).

### 2.5. In Vitro Bioactivity Analysis

The apatite formation activities of the nHAP/CS, Y-nHAP/CS, Eu-nHAP/CS, and Eu,Y-nHAP/CS specimens were analyzed by monitoring their morphological changes after immersing them in simulated body fluid (SBF). For the bioactivity analysis, the SBF solution with a similar ionic concentration as human blood plasma was prepared using the Kokubo method [46]. In this analysis, the prepared implant specimens were immersed in the SBF solution at 310 K in a DI water bath for 14 days. After that, the samples were washed with DI water to clean the surface and dried at 60 °C for 2 h. The bioactivity was confirmed through observing the calcium phosphate layer on the sample surface via FE-SEM measurements. The pH levels of the implant-specimen-socked physiological solutions were measured by a Thermo Orion pH meter using an ion-selective electrode. The pH level tests were performed in triplicate, and then the average values were recorded.

### 2.6. Nanoindentation Analysis

The mechanical properties of the samples were examined by the nanoindentation method analyzed using a TriboIndenter system (Hysitron, Minneapolis, MN, USA) equipped with a Berkovich diamond indenter. The load–displacement profiles were continuously recorded with a higher applied peak load of 1000 µN. The applied load was held for 10 s and then unloaded with a ratio of 0.2 s µN^−1^. The hardness (*H*_S_) and Young’s modulus (*E*_Y_) values of the samples were determined by means of the Oliver–Pharr relation using the tangent slopes obtained from the indentation analysis data.

### 2.7. In Vitro Antibacterial Activity

The antibacterial resistances of the samples were evaluated through the well diffusion method that utilizes the gram-positive (*E. coli*) and gram-negative (*B. subtilis*) human pathogenic microorganisms [37]. For this analysis, initially, the agar–agar was dissolved and poured into a petri dish with a thickness of 4 mm. Then, to achieve a semi-affluent growth of the organisms, the micro-organisms were inoculated on the solidified agar medium as a dense layer. For assessing the antibacterial activity, the 4 mm well was positioned at an identical distance, and then 50 μg/mL of nHAP/CS, Y-nHAP/CS, Eu-nHAP/CS, and Eu,Y-nHAP/CS biocomposites were individually loaded and hatched at 37 °C for 24 h. Subsequently, the antibacterial activity was examined by observing the bacterial inhibition zone around the well.

### 2.8. In Vitro Cell Culture Studies

Mouse embryonic fibroblasts (NIH 3T3, NCCS, Pune, India), which had been supplemented with 10% fetal bovine serum, were detached with trypsin-EDTA to obtain a single-cell suspension. The cells were seeded in each well at the density of 1 × 10^4^ cells per well. For the cell attachment, the cells were incubated under a CO_2_ (5%) atmosphere at 37 °C. To determine the cell viability, the biocomposite samples (50 μg/mL) in a serum-free medium were loaded onto the wells. After 48 h of incubation, the tetrazolium salt solution (MTT, 3-[4,5-dimethylthiazol-2-yl]-2,5-diphenyltetrazolium bromide) was incubated at 37 °C for 4 h. The MTT was reduced to an insoluble formazan, and the formed formazan crystals were solubilized using dimethyl sulfoxide. The absorbance was observed, and the percentage of cell viability was measured using the standardization of optical densities (ODs) to the negative controls.

## 3. Results and Discussion

**Figure 1:**
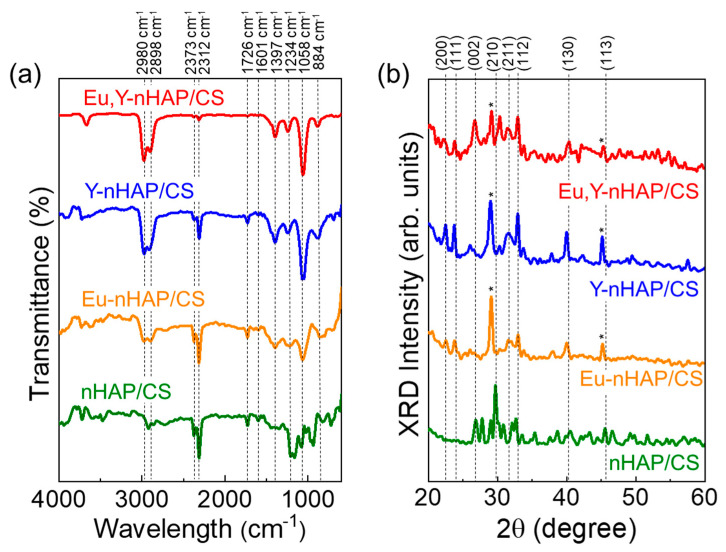
(**a**) FTIR spectra and (**b**) XRD patterns of the nHAP/CS, Eu-nHAP/CS, Y-nHAP/CS, and Eu,Y-nHAP/CS biocomposites.

Figure 1a shows the FTIR spectra of the nHAP/CS, Eu-nHAP/CS, Y-nHAP/CS, and Eu,Y-nHAP/CS biocomposites. For nHAP/CS, the sample displayed ten specific absorption peaks, which were closely relevant to the CS-hybridized nHAP composite system. 

The peaks at 884 and 1058 cm^−1^ were attributable to the ν_1_ and ν_3_ bending vibrations of the PO_4_^3−^ groups in the HAP, respectively [47]. The absorption peaks at 1234, 1397, 1601, and 1726 cm^−1^ were associated with the vibration modes from the CN–NH, CH_2_–OH, CH_3_, –NH, –NH_2_, and amide I carboxyl groups [48,49]. The peaks at the 2312 and 2373 cm^−1^ absorption bands arose from the CO_3_^2−^ stretching vibrations [50,51,52], and the other peaks observed at 2898 and 2980 cm^−1^ originated from the C–H stretching vibration for CS [53]. Such a clear observation of P-, C-, and N-related hybridized bonds and functional groups obviously depicted that effective nHAP/CS hybridization occurred in the prepared composite system. When adding the Eu and Y substituents into nHAP/CS (i.e., Eu-nHAP/CS, Y-nHAP/CS, and Eu,Y-nHAP/CS), the absorption peak intensities at 1234, 1726, 2312, and 2373 cm^−1^ decreased, whereas those at 1058, 1397, 2898, and 2980 cm^−1^ increased. This was presumably due to the substitution of the ions in the nHAP/CS through the composite formation.

Next, the crystallographic properties were investigated through XRD measurements. As shown in Figure 1b, all the prepared samples revealed the polycrystalline phases of the apatite. Namely, the samples exhibited the predominant XRD patterns at 22.26, 23.83, 26.66, 29.13, 31.54, 33.06, 39.94, and 45.15°, which corresponded to the hexagonal (200), (111), (002), (210), (211), (112), (130), and (113) phases of the crystalline apatite, respectively (JCPDS no.: 9-0432) [54,55,56]. Here, one can find that, as indicated by star marks, the doped samples (i.e., Eu-nHAP/CS, Y-nHAP/CS, and Eu,Y-nHAP/CS) showed the Bragg angle shift of the (210) and (113) phases toward the lower 2θ region than those of the nHAP/CS. This might be ascribed to the reorientation of apatite lattices because the ionic substitution would lead to the morphological evolution of local crystal lattices. Despite the Bragg angle shift, however, no secondary phases appeared in the doped samples. Additionally, when compared with bare nHAP samples with no CS (Figure S1), one can also confirm that there were no secondary phases in the nHAP-CS hybridized composites. Therefore, one can conjecture that no secondary phase particles were formed inside the doped nHAP/CS samples while the Eu and/or Y dopants were effectively incorporated into the host lattices of the nHAP/CS.

**Figure 2:**
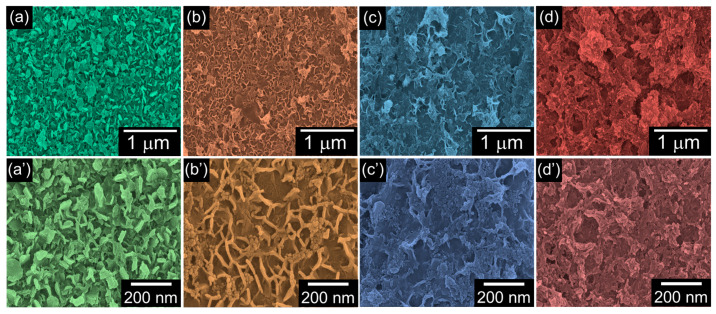
FE-SEM images of the (**a**,**a’**) nHAP/CS, (**b**,**b’**) Eu-nHAP/CS, (**c**,**c’**) Y-nHAP/CS, and (**d**,**d’**) Eu,Y-nHAP/CS composites. The upper and the lower panels are the low- and high-magnification images, respectively.

To monitor the morphological evolution of the samples, SEM measurements were carried out. Figure 2 shows the SEM images of four different 316L SS implant specimens, onto which nHAP/CS, Eu-nHAP/CS, Y-nHAP/CS, and Eu,Y-nHAP/CS biocomposites were coated. 

In the case of the bare nHAP/CS (Figure 2a,a’), the sample exhibited a porous microstructure with aggregated HAP nanoflakes. The average flake size was approximately ~30 nm (Figure 2a), and the HAP nanoflakes were well blended in the fibrous CS matrix (Figure 2a’). For the Eu-nHAP/CS (Figure 2b,b’), the sample displayed laterally elongated flakes with a highly microporous texture. In the case of the Y-nHAP/CS (Figure 2c,c’), the sample revealed intergranular grain aggregation. Accordingly, in the Eu,Y-nHAP/CS (Figure 2d,d’), a mixture of features from both Eu-nHAP/CS and Y-nHAP/CS were observed. Namely, the intergranular HAP flakes were densely aggregated and elongated along the lateral surface directions. Owing to the increased intergranular texture, the Eu,Y-nHAP/CS sample showed a high density of pores, which are beneficial for biological activity because the high porosity may help to increase the interaction between the composites and cells during implantation.

**Figure 3:**
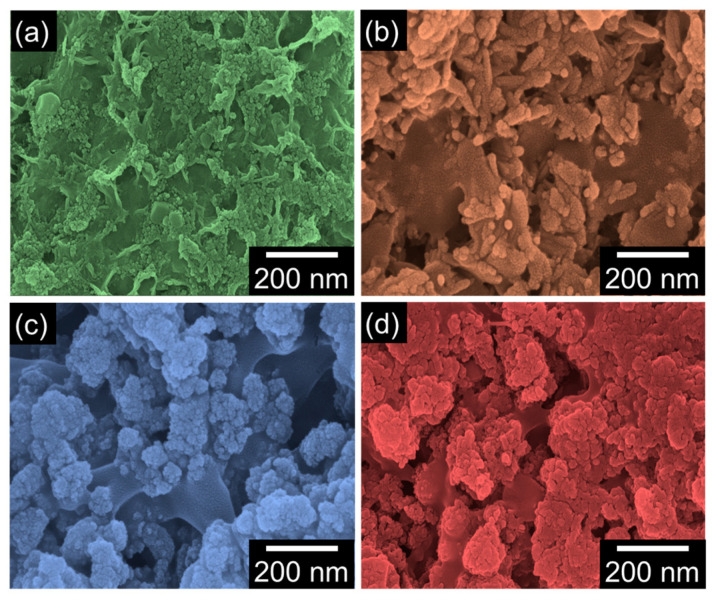
FE-SEM images of the SBF-immersed biocomposites for the in vitro bioactivity test: (**a**) nHAP/CS, (**b**) Eu-nHAP/CS, (**c**) Y-nHAP/CS, and (**d**) Eu,Y-nHAP/CS.

To examine the biological activity of the synthesized biocomposites, the in vitro bioactivities were assessed via observing the morphological changes after immersing the biocomposite-coated specimens in SBF for 21 days. In comparison with the as-synthesized states (Figure 2), the SBF-immersed samples exhibited modified surface features (Figure 3). Namely, one can find that long-term SBF immersing led to the deposition of the spherical apatite grains onto the biocomposite surfaces. For example, the nHAP/CS surface was entirely covered with agglomerated apatite grains (Figure 3a). Additionally, new granular-like textures also arose in the SBF-immersed nHAP/CS. These features were also observable in the other samples of Eu-nHAP/CS (Figure 3b), Y-nHAP/CS (Figure 3c), and Eu,Y-nHAP/CS (Figure 3d). Furthermore, the SBF-immersed samples did not display any cracks on their surfaces. This indicates that the samples possessed highly stable surface morphologies for apatite coating even after SBF immersing for 21 days. Particularly, one can confirm that the Eu,Y-nHAP/CS sample revealed an enhanced apatite deposition activity (i.e., full coverage of apatite grains onto the entire Eu,Y-nHAP/CS surface area). We attribute such a superior apatite deposition activity of Eu,Y-nHAP/CS to its high density of micropores because they could act as the nucleation sites for forming the apatite grains.

**Figure 4:**
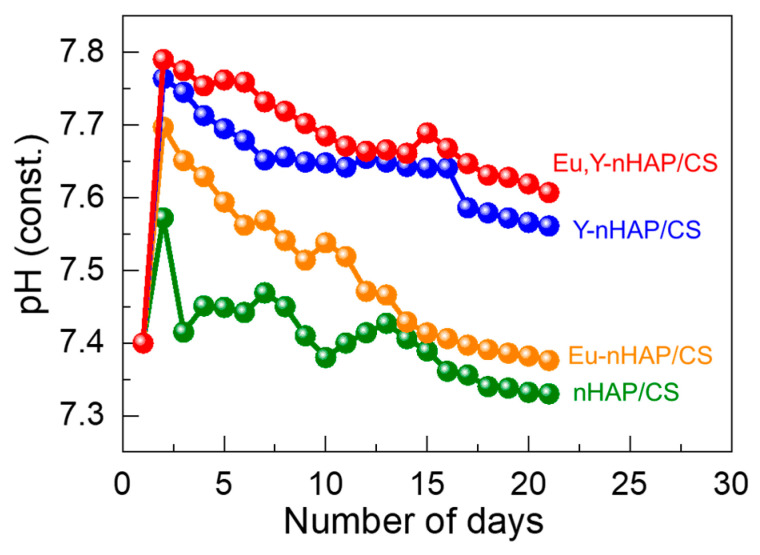
Comparison of the pH levels for the nHAP/CS, Eu-nHAP/CS, Y-nHAP/CS, and Eu,Y-nHAP/CS composites immersed in SBF for 21 days.

The in vitro bioactivity can also be examined by observing the pH level after immersing the target sample into the SBF solution. Thus, the pH levels of the SBF-immersed specimens were tested at 1-day intervals for 21 days. Here, we noted that the pH variation was recorded using the ion-selective electrode method. As shown in Figure 4, during SBF immersion, the pH level rapidly increased after 1 day. We believe such behavior was caused by the initial release of excessive PO_4_^3−^ from the host nHAP/CS material. After the initial increase in pH, the pH levels monotonically decreased with an increasing number of SBF immersion days. Although all four different specimens exhibited the same feature, the Eu,Y-nHAP/CS sample maintained its higher pH level compared with those of other samples. Since the pH deviation rate relied on the insolubility of the biocomposite, the higher pH level of Eu,Y-nHAP/CS verified its higher solubility. Correspondingly, the high solubility of Eu,Y-nHAP/CS further corroborated its enhanced apatite deposition activity, because both its high solubility and porosity were favorable for increasing the ionic migration and deposition rates at the surface medium of the biocomposite.

**Figure 5:**
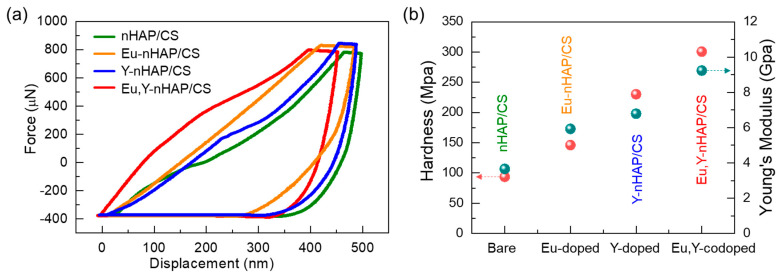
(**a**) Load–displacement indentation curves of the nHAP/CS, Eu-nHAP/CS, Y-nHAP/CS, and Eu,Y-nHAP/CS composites. (**b**) Hardness and Young’s modulus for the same samples.

For clinical applications of nHAP, mechanical strength is also of great importance. Thus, we assessed the mechanical properties of the prepared specimens with nanoindentation measurements. Figure 5a shows the load–displacement curves at a high load (1000 μN) for the nHAP/CS, Eu-nHAP/CS, Y-nHAP/CS, and Eu,Y-nHAP/CS samples. From the indentation curves, it was observable that the mineral incorporation led to significant changes in both the loading behavior and mechanical integrity. At the maximum load, the penetration depth of the indenter tip was 523, 511, 468, and 475 nm for the nHAP/CS, Eu-nHAP/CS, Y-nHAP/CS, and Eu,Y-nHAP/CS samples, respectively. 

To quantitatively examine and compare the mechanical strengths of the prepared specimens, the *H*_S_ and *E*_Y_ values were determined from the indentation curves. For nHAP/CS, the *H*_S_ and *E*_Y_ were estimated to be 93.57 MPa and 3.66 GPa, respectively, and these values were observed to increase when incorporating the mineral substituents (Figure 5b). Among them, Eu,Y-nHAP/CS showed to have higher values of *H*_S_ (300.71 MPa) and *E*_Y_ (9.24 GPa) compared with those of the other samples. This suggests that a surface-protective bioceramic coating could improve the mechanical strength of the load-bearing implant.

**Figure 6:**
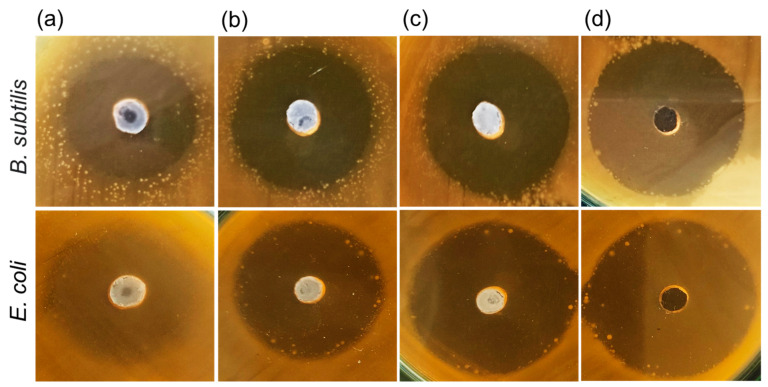
Antibacterial activities of the (**a**) nHAP/CS, (**b**) Eu-nHAP/CS, (**c**) Y-nHAP/CS, and (**d**) Eu,Y-nHAP/CS biocomposites against the bacterial strains of *B. subtilis* (upper panels) and *E. coli* (lower panels).

For more clarity on the potential applications of the present biocomposites, we examined their antibacterial characteristics by means of the well diffusion technique. Figure 6 displays the antibacterial activities of the biocomposite-coated implant specimens against the gram-negative (*E. coli*) and gram-positive (*B. subtilis*) bacterial strains. We here note that, for all the test samples, an identical amount of the 50 µL bacterial strain was used. In the case of the pristine nHAP/CS (Figure 6a), the inhibition zones were created around the wells in both *E. coli* and *B. subtilis*. However, the sample exhibited unclear microbial inhibitory zones, which was indicative of the relatively weak bacterial resistance. Similarly, the microbial inhibitory zones were also unclear for Eu-nHAP/CS (Figure 6b) and Y-nHAP/CS (Figure 6c). In the case of Eu,Y-nHAP/CS (Figure 6d), however, the sample displayed enhanced antibacterial activity against both the pathogenic organisms. Namely, the microbial inhibitory zones clearly appeared in both the *E. coli* and *B. subtilis* cases. Such a supreme antibacterial activity of Eu,Y-nHAP/CS was thought to result from the increased positive surface charges in Eu,Y-nHAP/CS.

**Figure 7:**
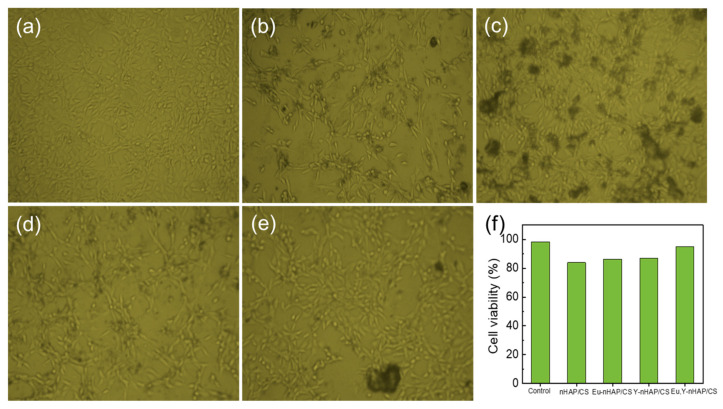
Optical microscopy images (magnification: ×20) of (**a**) control, (**b**) nHAP/CS, (**c**) Eu-nHAP/CS, (**d**) Y-nHAP/CS, and (**e**) Eu,Y-nHAP/CS composites treated with the NIH 3T3 cell line and (**f**) cell viability of the samples.

Next, the cytotoxicity of the prepared biocomposites was analyzed using the MTT assay method. Figure 7a–e show the optical microscopy images of the samples that were treated with a NIH 3T3 cell line. Here, we note that 50 μg/mL of the biocomposites were used. All the images represent the nontoxic nature of the biocomposites. Namely, all the samples showed a similar aspect that represented the fibroblast cell-growth behavior. Among them, Eu,Y-nHAP/CS showed better cell viability (94.9%) than the others (84–87%) (Figure 7f). The Eu,Y-nHAP/CS composites were soaked in the culture medium of the NIH 3T3 cell line. A lower cytotoxic effect in Eu,Y-nHAP/CS can be interpreted in terms of the enhanced live cell rate, attributable to the increased nucleation sites of the biocompatible composite.

**Figure 8:**
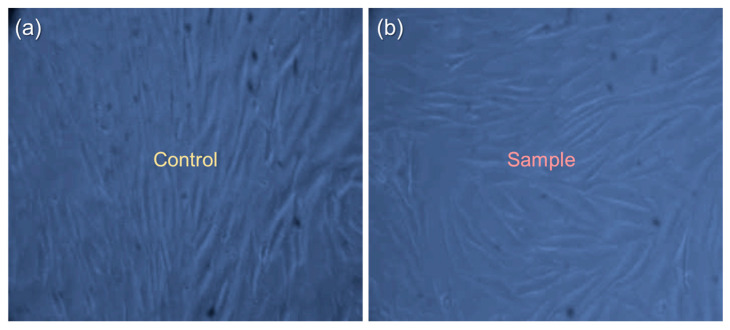
Optical microscopy images (magnification: ×20) of the live/dead cell assay measured through the Trypan blue staining method for the (**a**) control and (**b**) Eu,Y-nHAP/CS biocomposites seeded with the MG63 cell.

After confirming the superior characteristics of Eu,Y-nHAP/CS compared with the other materials, finally, the live/dead cell assay was evaluated for the Eu,Y-nHAP/CS-coated 316L SS implant specimen. To monitor the presence of live and dead cells, the Trypan blue staining method was utilized using the MG 63 cell. First, the MG 63 cells were seeded onto the Eu,Y-nHAP/CS surface for 48 h. After 72 min of incubation at 37 °C, the sample was stained with Trypan blue, then the seeded cells on the Eu,Y-nHAP/CS were viewed through an optical microscope. As shown in Figure 8a,b, the control and Eu,Y-nHAP/CS samples clearly exhibited macrophage-like raw cell lines with no dead cells in the culture vessels. This obviously specified that the Eu,Y-nHAP/CS biocomposite was nontoxic to the cell culture.

## 4. Conclusions

The high-quality Eu,Y-nHAP/CS biocomposite was synthesized through the facile wet precipitation technique using the cost-effective and environmentally friendly biomass waste of eggshells. The Eu,Y-nHAP/CS biocomposite exhibited a highly porous texture with an intergranular morphology, where the polycrystalline apatite grains were densely aggregated. When using Eu,Y-nHAP/CS as a bioceramic for biological and clinical implant applications, the sample clearly revealed a high antibacterial activity against both *E. coli* and *B. subtilis* bacterial strains. Furthermore, Eu,Y-nHAP/CS exhibited low cytotoxicity and high cell viability (94.9%) because of its high activity to grow bone-like active apatite sites. In addition, Eu,Y-nHAP/CS showed to possess a high mechanical strength (i.e., *E*_Y_ = 9.24 GPa and *H*_S_ = 300.71 MPa). These results suggested that biomass-derived Eu,Y-nHAP/CS is affordable as a potential candidate for a biomedical orthopedic implant application.

## Data Availability

The data presented in this study are available on request from the corresponding author.

## Supplementary Materials

The following supporting information can be downloaded at: https://www.mdpi.com/article/10.3390/nano12234302/s1, Figure S1: XRD patterns of nHAP, Eu-nHAP, Y-nHAP, and Eu,Y-nHAP.
