# Supplementary material for: In Situ Facile Synthesis of Low-Cost Biogenic Eggshell-Derived Nanohydroxyapatite/Chitosan Biocomposites for Orthopedic Implant Applications
Source: Nanomaterials (Basel). 2022 Dec 4;12(23):4302. doi: 10.3390/nano12234302 (PMC9739235; doi:10.3390/nano12234302)
Supplement: Supplementary file 1 [file nanomaterials-12-04302-s001.zip › nanomaterials-2043091-supplementary.pdf]

nanomaterials

Supplementary Materials

In Situ Facile Synthesis of Low-Cost Biogenic Eggshell-Derived Nanohydroxyapatite/Chitosan Biocomposites for Orthopedic Implant Applications

Sankar Sekar [1,2] and Sejoon Lee [1,2,*]

[1] Department of Semiconductor Science, Dongguk University-Seoul, Seoul 04620, Republic of Korea
[2] Quantum-Functional Semiconductor Research Center, Dongguk University-Seoul, Seoul 04620, Republic of Korea
* Correspondence: sejoon@dongguk.edu

Citation: Sekar, S.; Lee, S. In Situ Facile Synthesis of Low-Cost Biogenic Eggshell-Derived Nanohydroxyapatite/Chitosan Biocomposites for Orthopedic Implant Applications. *Nanomaterials* **2022**, *12*, 4302. https://doi.org/10.3390/nano12234302

Academic Editors: Vanessa Valdiglesias and Blanca Laffon

Received: 3 November 2022
Accepted: 29 November 2022
Published: 4 December 2022

Publisher's Note: MDPI stays neutral with regard to jurisdictional claims in published maps and institutional affiliations.

Copyright: © 2022 by the authors. Licensee MDPI, Basel, Switzerland. This article is an open access article distributed under the terms and conditions of the Creative Commons Attribution (CC BY) license (https://creativecommons.org/licenses/by/4.0/).

Structural Characteristics of Undoped and Doped nHAP Samples

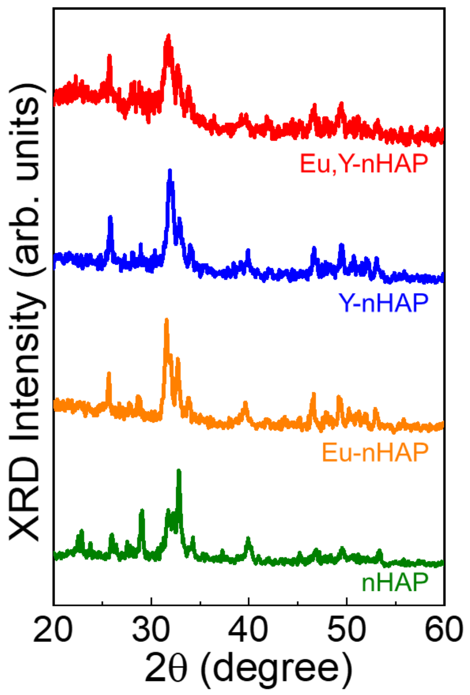

Figure S1. XRD patterns of nHAP, Eu-nHAP, Y-nHAP, and Eu,Y-nHAP.
